# *Pseudomonas* Infection Outbreak Associated with a Hotel Swimming Pool — Maine, March 2023

**DOI:** 10.15585/mmwr.mm7302a2

**Published:** 2024-01-18

**Authors:** Liz Lamere, Emer Smith, Heather Grieser, Matthew Arduino, Michele C. Hlavsa, Stephen Combes

**Affiliations:** ^1^Epidemic Intelligence Service, CDC; ^2^Maine Center for Disease Control and Prevention; ^3^MCD Global Health, Hallowell, Maine; ^4^Division of Healthcare Quality Promotion, National Center for Emerging and Zoonotic Infectious Diseases, CDC; ^5^Division of Foodborne, Waterborne, and Environmental Diseases, National Center for Emerging and Zoonotic Infectious Diseases, CDC.

**Summary**

**What is already known about this topic?**

Treated recreational water venues (e.g., pools and hot tubs) located at hotels or resorts represent one third of sources of reported outbreaks associated with treated recreational water.

**What is added by this report?**

In March 2023, 23 persons developed ear pain, rash, or pain or swelling in their feet or hands after swimming in a hotel pool in Maine. The outbreak was caused by *Pseudomonas aeruginosa*. Inadequate maintenance and monitoring of chlorine concentration likely contributed to this outbreak.

**What are the implications for public health practice?**

Outbreak prevention strategies include maintaining chlorine concentration and otherwise vigilantly managing the pool, especially during January–April, and disseminating prevention messaging to pool and hot tub users.

## Abstract

Treated recreational water venues (e.g., pools and hot tubs) located at hotels represent one third of sources of reported treated recreational water–associated outbreaks; when these outbreaks are caused by *Pseudomonas aeruginosa*, they predominantly occur during January–April. On March 8, 2023, the Maine Center for Disease Control and Prevention (Maine CDC) initiated an investigation in response to reports of illness among persons who had used a swimming pool at hotel A during March 4–5. A questionnaire was distributed to guests who were at hotel A during March 1–7. Among 35 guests who responded, 23 (66%) developed ear pain, rash, or pain or swelling in feet or hands within days of using the pool during March 4–5. *P. aeruginosa*, a chlorine-susceptible bacterium, was identified in cultures obtained from skin lesions of three patients; a difference of two single nucleotide polymorphisms was found between isolates from two patients’ specimens, suggesting a common exposure. Hotel A management voluntarily closed the pool, and Maine CDC’s Health Inspection Program identified multiple violations, including having no disinfectant feeder system, all of which had been identified during a previous inspection. Because chlorine had been added to the pool water after the pool was voluntary closed, environmental samples were not collected. The pool remained closed until violations were addressed. Health departments can play an important role in reducing the risk for outbreaks associated with hotel pools and hot tubs. This reduction in risk can be achieved by collaborating with operators to ensure compliance with public health codes, including maintaining chlorine concentration and otherwise vigilantly managing the pool, and by disseminating prevention messages to pool and hot tub users.

## Investigation and Results

### Reports of Illnesses Associated with Hotel A Swimming Pool

On March 7, 2023, the Maine Center for Disease Control and Prevention (Maine CDC) received a report from a group of hotel guests who had developed ear pain, rashes, and eye irritation after using a swimming pool at hotel A during the March 4–5 weekend. Later that day, an additional family that had used hotel A’s swimming pool the same weekend reported experiencing similar illness, in addition to redness and pain of their hands and feet. Over the next 2 days, four additional groups of guests reported similar illnesses after using the hotel A pool during the same weekend. On March 7, in response to the reports, Maine CDC’s Health Inspection Program contacted hotel A and learned that management had voluntarily closed the pool after receiving guest complaints. In response, on March 8, Maine CDC initiated an epidemiologic, laboratory, and environmental health investigation.

### Case Identification

Maine CDC used illness reports to identify six households with members who had visited hotel A’s pool during the weekend of March 4–5 and interviewed a representative from each household using a standard questionnaire. Interviewers asked about other groups or persons who had used the hotel pool; additional identified persons were also contacted and interviewed. Guests were asked about their use of the hotel A pool, other pools, and hot tubs since February 24, and subsequent illness.

**FIGURE 1:**
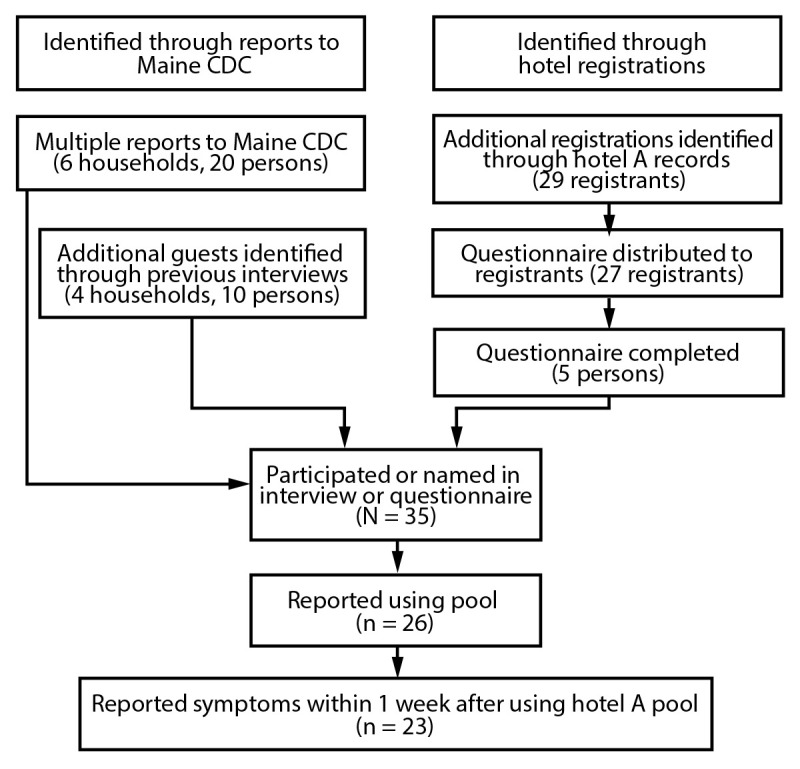
Identification of guests who used the pool at hotel A — Maine, March 4–5, 2023*^,†^ **Abbreviation:** Maine CDC = Maine Center for Disease Control and Prevention. * “Additional registrations” are registration records for guests not previously identified; records did not include the total number of guests per registration. ^†^ The survey questionnaire was not distributed to two of the 29 hotel A registrants; these two guests booked through a third-party website and did not input a telephone number or email address.

A total of 10 households (30 guests) were identified as having visited or used the hotel A pool during March 4–5 (Figure 1); one person per household was interviewed about all household members who used the pool. To further assess the scope of the outbreak, Maine CDC obtained a list of registered guests who were at hotel A during March 1–7 and identified 29 additional registrants who might have been present at hotel A during March 4–5. These additional registrants were sent the standardized questionnaire by text or email and asked to fill out a separate questionnaire for each household member who used the pool. Questionnaires were completed for five additional guests.

A total of 15 interviews or questionnaires were completed for 35 unique persons. Maine CDC requested that symptomatic guests ask their health care provider to obtain a skin lesion swab for laboratory analysis and requested that laboratories send any isolates to Maine’s Health and Environmental Testing Laboratory. This study was reviewed by CDC, deemed not research, and was conducted consistent with applicable federal law and CDC policy.*

**FIGURE 2:**
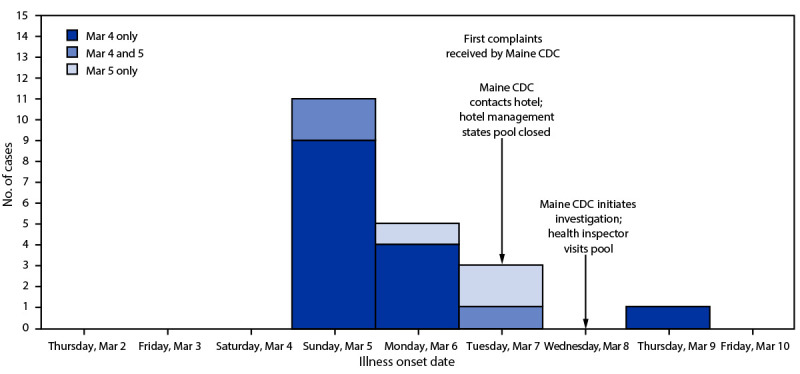
Identified cases of *Pseudomonas* infection, by dates of illness onset and hotel swimming pool use (n = 20)* — Maine, March 2023 **Abbreviation:** Maine CDC = Maine Center for Disease Control and Prevention. * Illness onset date not available for three pool users with reported illness.

A case was defined as the occurrence of ear pain, rash, or pain or swelling in feet or hands in a person within 7 days after using the hotel pool during March 4–5. Among 35 persons for whom information was available, 26 (74%) reportedly used the hotel A swimming pool during March 4–5. Among these 26 persons, 23 (88%) experienced an illness meeting the case definition; illness onset date was available for 20 persons (Figure 2). Among the 23 patients, 16 (70%) had ear pain, 15 (65%) had a rash, and seven (30%) had pain or swelling in their feet or hands (Table). Fifteen (65%) patients were female. Among 22 patients with available information, age ranged from 5 months to 61 years (median = 8 years). Among 20 patients with reported time and date of illness onset, illness began a median of 24 hours (range = 8 hours–6 days) after use of the hotel A pool.

**TABLE:** Characteristics of illness and exposure to swimming pool water — hotel A, Maine, March 2023

Characteristic	No. of cases (%)
**Total**	**23 (100)**
**Time of illness onset after pool use, hrs**	
<12	1 (4)
12–23	5 (22)
24–48	13 (57)
>48	1 (4)
Unknown	3 (13)
**Signs and symptoms**	
Ear pain	16 (70)
Rash	15 (65)
Runny nose	9 (39)
Pain or swelling in hands or feet	7 (30)
Eye irritation	5 (22)
Fatigue	4 (17)
Diarrhea	3 (13)
Joint pain	2 (9)
Lymphadenopathy	2 (9)
Vomiting	2 (9)
**Length of time spent in pool, hrs**	
<1	4 (17)
1–2	12 (52)
>2	3 (13)
Unknown	4 (17)
**Interval from pool use to showering after pool use, hrs**	
<1	2 (9)
>1, same day	3 (13)
No shower same day	4 (17)
Unknown or no response	14 (61)

### Laboratory Evaluation

Skin lesion swabs were obtained from three patients, two of whom were family members who lived in separate households, spent time together outside of the hotel pool, and had no other pool or hot tub exposures. *P. aeruginosa* was identified in all three specimens; the isolates of the two family members were sent to Maine’s Health and Environmental Testing Laboratory for whole genome sequencing. Single nucleotide polymorphism (SNP) analysis, using CLC-BIO (version 23.0.2; Qiagen Aarhus), indicated that the two isolates were highly related with a two-SNP difference, suggesting a common exposure.

### Hotel A Pool Inspections

In January 2022, the hotel pool had failed a routine health department inspection. During that inspection, several violations were identified: 1) no operator had successfully completed approved training, 2) no pool logs documenting free chlorine^†^ concentration readings at least three times per day while the pool was open for use, 3) no posted routine operating procedures, and 4) no functioning disinfectant feeder installed. During the subsequent March 8, 2023, inspection, the health inspector noted that although the hotel did have an operator who had successfully completed approved training <2 weeks before inspection, none of the other previously identified violations had been corrected. The pool logs for March 1–5 showed two compliant free chlorine concentration readings, not the expected 15, and both readings were dated March 3. The inspector also noted that an indeterminant amount of chlorine had been added to the pool water by hotel staff members after they voluntarily closed the pool; therefore, water quality and environmental samples were not collected.

Because the identified violations included imminent health hazards and uncorrected previously identified violations, Maine CDC’s Health Inspection Program directed hotel A to not reopen the pool until all the violations were addressed. The health inspector provided recommendations to address the violations. On reinspection 1 month later, the violations were noted to be corrected. No additional hotel A pool-associated illnesses were identified after reopening.

## Discussion

*P. aeruginosa* can cause acute otitis externa (swimmer’s ear), folliculitis (hot tub rash) (*1*), and painful nodular lesions on the soles or palms (hot hand-foot syndrome) (*2*) and is likely to be transmitted through contact with contaminated water in pools or hot tubs and not through person-to-person contact (*3*). *P. aeruginosa* is readily inactivated by disinfectants such as chlorine and bromine. Because of this, maintaining a minimum free chlorine concentration of at least 1 ppm^§^ in treated recreational water venues open to the public as recommended by CDC and as required by Maine’s pool code, prevents waterborne transmission of most pathogens, including *P. aeruginosa*. The lack of an installed and functioning disinfectant feeder in this pool and inadequate monitoring of the free chlorine concentration during March 1–5, particularly the March 4–5 weekend, would make it more challenging to maintain adequate free chlorine concentration, and thus, more challenging to prevent pathogen transmission. Inadequately maintained disinfectant concentration can lead to proliferation of *P. aeruginosa* and buildup of biofilm on wet venue surface, scale, and sediment. Biofilm is a primarily polysaccharide matrix that is produced by microbial cells and in which bacteria are embedded; biofilm is difficult to remove and cannot be removed by gentle rinsing (*4*). Even when adequate disinfectant concentration is maintained, the extracellular matrix of the biofilm can protect *P. aeruginosa* and other pathogens from disinfectants.

Among 987 treated recreational water–associated outbreaks reported to CDC for the period 1971–2021, 369 (37.4%) were linked to a hotel setting (i.e., hotel, motel, lodging, inn, or resort) (*5*). In addition, for the period 1971–2021, 38 states reported 222 outbreaks associated with treated recreational water venues that were confirmed or suspected to be caused by *P. aeruginosa* (*5*), 152 (68%) of which were associated with a hotel setting. Seventeen (11%) of these 152 outbreaks were associated with pools only and 87 (57%) with hot tubs only.^¶^ Among the 152 outbreaks, 100 (66%) began during January–April. Outbreak exposures often occur during a weekend, when trained operators might not be on duty, and when events, including parties and sports tournaments, are scheduled (*6*).

To help prevent outbreaks caused by *P. aeruginosa* and other pathogens readily inactivated by disinfectants, local, state, territorial, and tribal jurisdictions can voluntarily adopt recommendations in CDC’s Model Aquatic Health Code (MAHC).** Such recommendations include having an operator who has successfully completed approved training to ensure adequate recreational water disinfectant concentration (MAHC 5.7.3.1.1 and 5.7.3.1.2) (*7*,*8*) and conducting a daily preopening inspection for biofilm (MAHC 6.4.1.3.1) and, if needed, removing biofilm by vigorous scrubbing. Public health officials can also increase awareness of healthy swimming by disseminating prevention messages, including recommendations to check the latest inspection score before using the pool. Much like restaurant inspection scores, scores from inspections of treated recreational water venues open to the public provide an assessment of operation and management. These scores are often posted waterside or on the jurisdiction’s website. Because inspections are a snapshot in time, pool and hot tub users can additionally protect themselves by conducting their own mini-inspection before getting in the water^††^(e.g., measuring the disinfectant levels and pH using test strips that are readily available at hardware and big-box stores); such interventions should not replace pool and hot tub management but can provide users a timely assessment of water conditions.

### Limitations

The findings in this report are subject to at least two limitations. First, the reported number of persons who used the hotel pool and subsequently developed illness meeting the case definition likely underestimates the actual incidence. For example, although the hotel identified 29 additional guest registrants who were at the hotel during the March 4–5 weekend, only five completed the questionnaire. Second, a definitive link between illness and the hotel pool could not be established. The two isolates found to be only two SNPs apart were from family members who both used the hotel pool but also spent time together outside of the hotel pool. Not collecting environmental samples precluded molecular characterization of an isolate and comparison with clinical isolates. However, neither family member reported other pool or hot tub exposures, and *P. aeruginosa* is likely to be transmitted through contaminated water but not person to person. The hotel pool remains the most likely source of exposure.

### Implications for Public Health Practice

Enforcement of local, state, territorial, and tribal codes and dissemination of prevention messaging to pool and hot tub users can reduce the likelihood of outbreaks caused by *P. aeruginosa* and other pathogens. Healthy swimming promotion efforts are especially necessary when the public might be more likely to stay at hotels and use the pools and hot tubs. To prevent outbreaks, operators should be vigilant about proper operation and management, especially during January–April and weekends.

## Footnotes

* 45 C.F.R. part 46, 21 C.F.R. part 56; 42 U.S.C. Sect. 241(d); 5 U.S.C. Sect. 552a; 44 U.S.C. Sect. 3501 et seq.

† Free chlorine is the most active disinfectant form of chlorine.

§ At 1 ppm free chlorine, most pathogens are inactivated within minutes at a pH of 7.0–7.8 and temperature of 77°F (25°C). pH determines the relative amounts of hypochlorous acid (HOCl, the most active free chlorine disinfectant) and hypochlorite ion (OCl^–^, a less active free chlorine disinfectant).

¶ Another 44 (29%) outbreaks were associated with both pools and hot tubs; the remaining four (3%) were associated with other or unknown treated recreational water venues.

** For reference purposes, MAHC elements that could reduce the risk for illness or injury are discussed in this report are followed by the specific section number that covers that element.

††
https://www.cdc.gov/healthywater/swimming/materials/infographic-inspection.html
